# City-Level Sugar-Sweetened Beverage Taxes and Youth Body Mass Index Percentile

**DOI:** 10.1001/jamanetworkopen.2024.24822

**Published:** 2024-07-31

**Authors:** Deborah Rohm Young, Monique M. Hedderson, Margo A. Sidell, Catherine Lee, Deborah A. Cohen, Emily F. Liu, Lee J. Barton, Jennifer Falbe, Galina Inzhakova, Sneha Sridhar, Allison C. Voorhees, Bing Han

**Affiliations:** 1 Department of Research & Evaluation, Kaiser Permanente Southern California, Pasadena; 2 Division of Research, Kaiser Permanente Northern California, Oakland; 3 Department of Human Ecology, University of California, Davis

## Abstract

**Importance:**

Levying excise taxes on sugar-sweetened beverage (SSB) distributors, which are subsequently passed on to consumers, is a policy implemented to reduce the high prevalence of cardiometabolic disease and generate public health funding. Taxes are associated with lower SSB purchases and consumption, but it is unknown whether they are associated with weight-related outcomes in youth.

**Objective:**

To determine the association of SSB excise taxes with youth body mass index (BMI) trajectories.

**Design, Setting, and Participants:**

This cohort study was conducted from 2009 to 2020, including 6 years before tax implementation and 4 to 6 years after tax implementation. The California cities of Albany, Berkeley, Oakland, and San Francisco, which implemented SSB excise taxes, were compared against 40 demographically matched control cities in California. Participants included Kaiser Permanente members aged 2 to 19 years at cohort entry (baseline) with continuous residence in selected cities with at least 1 pretax and 1 posttax BMI recorded in their electronic health record. Data analysis was performed from January 2021 to May 2023.

**Exposure:**

Implementation of SSB excise taxes.

**Main Outcomes and Measures:**

Centers for Disease Control and Prevention age-specific and sex-specific BMI percentiles and percentage of youth with overweight or obesity before tax implementation through 4 to 6 years after implementation were compared with control cities. Statistical analysis was conducted using the difference-in-differences (DID) method. A sensitivity analysis used the synthetic control method.

**Results:**

A total of 44 771 youth (mean [SD] age at baseline, 6.4 [4.2] years; 22 337 female [49.9%]) resided in the cities with SSB taxes; 345 428 youth (mean [SD] age, 6.9 [4.2] years; 171 0168 female [49.5%]) resided in control cities. There was a −1.64–percentage point (95% CI, −3.10 to −0.17 percentage points) overall difference in the mean change of BMI percentile between exposure and control cities after SSB tax implementation. There was no significant overall difference in the percentage of youth with overweight or obesity or youth with obesity compared with control cities. All DID estimates were significant for youth residing in exposure cities in terms of BMI percentile (age 2-5 years in 2017, −2.06 percentage points [95% CI, −4.04 to −0.09 percentage points]; age 6-11 years in 2017, −2.79 percentage points [95% CI, −4.29 to −1.30 percentage points]), percentages of youth with overweight or obesity (age 2-5 years, −5.46 percentage points [95% CI, −8.47 to −2.44 percentage points]; age 6-11 years, −4.23 percentage points [95% CI, −6.90 to −1.57 percentage points]), and percentages of youth with obesity (age 2-5 years; −1.87 percentage points [95% CI, −3.36 to −0.38 percentage points]; age 6-11 years, −1.85 percentage points [95% CI, −3.46 to −0.24 percentage points]). Compared with control cities, changes in mean BMI percentiles were significant for male (−1.98 percentage points; 95% CI, −3.48 to −0.48 percentage points), Asian (−1.63 percentage points; 95% CI, −3.10 to −0.16 percentage points), and White (−2.58 percentage points; 95% CI, −4.11 to −1.10 percentage points) youth. Compared with control cities, White youth in exposure cities had improvements in the percentage with overweight or obesity (−3.73 percentage points; 95% CI, −6.11 to −1.35 percentage points) and the percentage with obesity (−2.78 percentage points; 95% CI, −4.18 to −1.37 percentage points).

**Conclusions and Relevance:**

In this cohort study, SSB excise taxes were associated with lower BMI percentile among youth. Policymakers should consider implementing SSB excise taxes to prevent or reduce youth overweight and obesity and, ultimately, chronic disease, particularly among children younger than 12 years.

## Key Points

**Question:**

Are city-level excise taxes on sugar-sweetened beverages (SSBs) associated with youth body mass index (BMI)?

**Findings:**

In this cohort study, 44 771 youth living in 4 California cities with SSB excise taxes had significant reductions in mean age-specific and sex-specific BMI percentiles after SSB tax implementation compared with mean changes in 345 428 youth living in control cities.

**Meaning:**

These findings suggest that policymakers should consider implementing SSB excise taxes to reduce the percentage of youth with high BMI percentiles, which may reduce prevalence of youth overweight and obesity.

## Teaser

This cohort study examines the association of sugar-sweetened beverage excise taxes with youth body mass index trajectories in 4 cities in California.

## Introduction

The prevalence of youth overweight or obesity status remains high: 16.1% of US youth had overweight and 19.3% had obesity in 2017 to 2018,^1^ increasing their risk of current and future chronic diseases.^2,3^ Although there are multilevel contributors to the obesity epidemic, it is clear that consumption of sugar-sweetened beverages (SSBs) is positively associated with obesity.^4^ Systematic reviews^5,6^ of SSB policies to reduce consumption among youth found mixed results but a trend for weight reduction, although studies’ quality were low. In 2019, the American Academy of Pediatrics recommended fiscal policies like SSB excise taxes to reduce SSB consumption.^7^

Berkeley, California, was the first US city to levy an SSB excise tax in 2015; currently, a total of 7 cities have implemented similar excise taxes, including other Bay Area cities of Albany, Oakland, and San Francisco, California. These cities levied a $0.01 per ounce excise tax onto SSB distributors. The excise taxes were passed on to consumers.^8,9,10^ SSB purchases^10,11^ and consumption^10,12,13,14^ declined in these cities, and revenues have funded public health and equity programs.^15^

Researchers have estimated the potential health outcomes of SSB excise taxes. At least 1 simulation study^16^ estimated that obesity prevalence in youth could be lowered by 1.6 to 2.4 percentage points over a 10-year period. In an observational study^17^ conducted in Mexico, which initiated a nationwide SSB tax, each 10% increase in SSB prices that followed the tax implementation was associated with a 1.3 percentage point decrease in overweight or obesity prevalence among adolescent girls but not boys. Using data from the Youth Risk Behavioral Surveillance System, Flynn^18,19^ found self-reported reductions in body mass index (BMI; calculated as weight in kilograms divided by height in meters squared) among high school students in the tax cities of Philadelphia, Pennsylvania; Oakland, California; and San Francisco, California; compared with controls. A study^20^ of the Philadelphia SSB tax found reduced tooth decay in adults and children enrolled in Medicaid. However, to our knowledge, the outcomes of SSB excise taxes on weight among youth across all age ranges in US cities has not been investigated. This information is vital for cities considering implementing SSB taxes to benefit community health and for policymakers as they consider which levers may be most effective in reducing the high prevalence of youth overweight and obesity.

We had the unparalleled opportunity at Kaiser Permanente (KP) Northern California and KP Southern California to conduct a rigorous, natural experiment to examine the outcomes of SSB excise taxes on child and adolescent BMI. We used existing data from electronic health records (EHRs) among patients living in the cities of Albany, Berkeley, Oakland, and San Francisco and 40 matched comparison cities. We examined BMI percentiles and percentage of youth with overweight or obesity before implementation of SSB taxes in these 4 cities through 4 to 6 years after implementation and compared with control cities across Northern and Southern California.

## Methods

### Study Design

This cohort study used a natural experiment design with an exposure and control group and evaluations before and after SSB excise tax implementation. The exposure group consisted of the 4 SSB excise tax cities, which are all located in the greater San Francisco area with distinct city and population characteristics. The control group consisted of 40 California cities without SSB excise taxes, which were chosen by matching each exposure city to 10 unique control cities without replacement. Control cities were selected differentially for each tax city. The goodness of matching between cities was based on a euclidean distance of standardized city-level variables (based on the 5-year averages of US Census American Community Survey starting 4 years before SSB implementation), including total population, population density, percentage female, age distribution, race and ethnicity, percentage of households in poverty, and distribution of educational status, as well as the percentage of population who were KP members. We excluded 3 control cities with good matching to tax cities because of their geographic proximity (ie, sharing a common border) with the exposure cities. Because of their different times of implementing the SSB excise tax,^21^ for Albany, Oakland, and San Francisco and their control cities, the pretax phase was 2010 to 2016 and the posttax phase was 2017 to 2020. For Berkeley and its 10 control cities, the pretax phase was 2009 to 2014 and the posttax phase was 2015 to 2020. The evaluation included the overall SSB tax outcomes across all 4 exposure cities and the entire posttax phase. Preplanned subgroups analyses were specified. Refer to the eAppendix and eTable 1 in Supplement 1 for more details of the city matching process.

### Data Source and Inclusion Criteria

This study is based on EHR data from the Northern California and Southern California regions of KP. KP is an integrated health care system with hospitals, medical offices, laboratories, and pharmacies that are linked by an information infrastructure that supports clinical practice and research needs. The EHR was fully implemented in 2007 and 2008. Between the 2 regions in California, KP serves more than 8.1 million patients, with demographics generally similar to the local population.^22,23^ The study was reviewed and approved by KP institutional review boards. The study followed the Strengthening the Reporting of Observational Studies in Epidemiology (STROBE) reporting guidelines for cohort studies. Informed consent was waived because the data are deidentified, in accordance with 45 CFR §46.

Inclusion criteria for study analyses were (1) having an active KP membership in at least 1 year during the study period and with primary residential addresses in study cities; and (2) having at least 1 record with an age between 2 and 19 years during the study period. We used the minimum age of 2 years given that toddlers and preschoolers consume SSBs,^24^ and simulation modeling suggests that parents of preschoolers are sensitive to SSB price increases from taxes.^25^ Exclusion criteria were any of the following health conditions: metastatic cancer, pregnancy, bariatric surgery in the last 5 years, and current palliative care. Patients having changed residence between an exposure city and 1 or more control cities during the study period were excluded.

Our main outcomes were age-specific and sex-specific BMI percentile and overweight and obesity status. For youth up to age 19 years, standing height and weight were assessed at each outpatient visit. The Centers for Disease Control and Prevention age-specific and sex-specific BMI percentiles were calculated and used to identify underweight (<5th percentile), healthy weight (5th-85th percentile), overweight (>85th percentile), and obesity (>95th percentile). Patients were categorized into the following age groups at cohort entry (baseline): 2 to 5, 6 to 11, and 12 to 19 years. A very few extreme values in original BMI values (≤5 or ≥91) were excluded (roughly 0.1%). Race and ethnicity were self-reported or reported by a primary caregiver and were categorized as non-Hispanic Asian or Pacific Islander (Asian or Pacific Islander), non-Hispanic Black or African American (Black), Hispanic, non-Hispanic White (White), and other (including American Indian or Alaska Native, multiracial, and other race and ethnicities not listed or unknown; these subgroups were combined owing to small sample sizes). Race and ethnicity were considered important variables to be considered because research indicates that Black and Hispanic youth are exposed to more SSB marketing^26^ and consume more SSBs than White youth.^27,28^ Previous studies suggested that SSB taxes would result in the greatest health gains for people with lower income and underrepresented racial and ethnic groups.^29^ Other covariates included insurance status (KP commercial plan vs Medicaid and all other options) and household poverty rate in the patient’s primary residential US Census tract.

The main analysis required an eligible patient to have at least 1 BMI percentile measurement in each of the 2 study phases. For a patient with more than 1 measurement within a calendar year, multiple measurements were averaged to form a patient-year BMI.

### Statistical Analysis

Data analysis was performed from January 2021 to May 2023. Our main analysis applied the difference-in-differences (DID) method, a well-established method to estimate the outcomes in natural experiments.^30,31^ Owing to the very large sample size, we applied a stratified analytic approach. The total sample was split into blocks that were defined jointly by exposure city, race and ethnicity, sex, age cohorts, and insurance status. There were approximately 100 blocks for each of the 3 outcomes, where the number and definitions of blocks varied among outcomes. Each block had an approximate maximum sample size of 50 000 patients or 100 000 records. Small blocks were combined with adjacent blocks with similar characteristics to form a minimum sample size of approximately 5000 patients or 10 000 records. In each block, we applied a saturated linear mixed-effect model with totally flexible temporal trends for all cities and a random effect for repeated measures within a patient. Placebo tests (ie, setting the last year in the pretax period as the posttax phase and removing the actual posttax phase)^30^ were conducted in each block to ascertain that the assumption of parallel trend in the exposure and control cities were not significantly violated. A significant placebo effect from such tests, if any, revealed the existence of pretreatment confounding that was not adequately accounted for by the analytic approaches. An insignificant placebo effect, although not ruling out all threats, improved the internal validity of the findings. Results from all block-level models were combined to form overall estimates, weighted by sample sizes of the exposure cities in each block. Preplanned subgroups analyses were by tax cities, years 1 to 4 after tax implementation, race and ethnicity, sex, age group (defined as age in 2017), overweight or obesity status, obesity status (in the pretax phase), and poverty status. The subgroup effect estimate was formed using the same aggregation method as in the overall effect estimate but limited to blocks belonging to the subgroup and can be interpreted as the effect limited to that subgroup. The DID analysis was conducted using SAS statistical software version 9.4 (SAS Institute). Refer to the eAppendix in Supplement 1 for more details of the DID approach.

In sensitivity analyses, we also applied the synthetic control method (SCM)^32^ to aggregated city-year level data to re-estimate the outcomes of SSB taxes and to compare with the results of the main analysis. The SCM is distinct from the DID method in fundamental causal inference assumptions and statistical inference procedures. The SCM used the sparse aggregated city-year level data (44 cities by 10 or 12 years) instead of patient-year EHR records. Although the SCM has relatively poor statistical efficiency, it offers easy visualization of outcome trajectories and suffices to assess the robustness of findings from the main analysis. The eAppendix in Supplement 1 provides further details of the sensitivity analysis. Statistical significance was set at 2-sided *P* < .05.

## Results

There were 328 candidate control cities within KP, of which 40 were selected for analysis, resulting in 44 cities (4 exposure and 40 controls). The matching of population-based demographics of each exposure city to the mean (SD) of its 10 control cities is displayed in Table 1. Although there was a discrepancy in population totals between the exposure cities and their controls because of the uniqueness of exposure cities, other demographic characteristics were similar. For instance, the percentage of female individuals was 51% in Albany, Berkeley, and Oakland, the same percentage as the mean of each of their 10 control cities. Age distribution was also similar (except for San Francisco, which tended to have fewer youth), as was membership in KP. The control cities of Oakland had fewer Black residents than the exposure city (12% vs 26%) and more Hispanic residents (43% vs 26%). Compared with the exposure cities, the control cities had fewer residents with college degrees or higher.

**Table 1.** Characteristics of Exposure and Control Cities Based on City-Level Data From the American Community Survey^a^

Characteristic	Albany		Berkeley		Oakland		San Francisco		Total	
	Exposure (n = 1)^b^	Controls (n = 10)^c^	Exposure (n = 1)^b^	Controls (n = 10)^c^	Exposure (n = 1)^b^	Controls (n = 10)^c^	Exposure (n = 1)^b^	Controls (n = 10)^c^	Exposure (N = 4)^c^	Controls (N = 40)^c^
Population										
Total per 10 000	1.91	6.12 (2.80)	1.13	7.26 (5.39)	40.49	19.03 (12.11)	8.46	47.96 (38.99)	34.59 (37.17)	20.09 (26.17)
Density, 1000 persons/mile^2^	3.50	3.94 (1.35)	6.41	5.84 (1.25)	5.20	4.18 (0.89)	3.65	4.92 (2.10)	4.69 (1.38)	4.72 (1.60)
KP membership	0.36	0.32 (0.06)	0.26	0.19 (0.05)	0.34	0.34 (0.09)	0.26	0.21 (0.08)	0.30 (0.05)	0.26 (0.09)
Sex										
Female	0.51	0.51 (0.01)	0.51	0.51 (0.01)	0.51	0.51 (0.01)	0.49	0.51 (0.01)	0.51 (0.01)	0.51 (0.01)
Male	0.49	0.49 (0.01)	0.49	0.49 (0.01)	0.49	0.49 (0.01)	0.51	0.49 (0.01)	0.49 (0.01)	0.49 (0.10)
Age group, y										
≤19	0.29	0.25 (0.04)	0.21	0.23 (0.05)	0.23	0.30 (0.04)	0.15	0.27 (0.04)	0.22 (0.06)	0.26 (0.05)
20-44	0.38	0.34 (0.05)	0.44	0.42 (0.06)	0.40	0.36 (0.02)	0.45	0.38 (0.02)	0.42 (0.03)	0.37 (0.05)
45-64	0.24	0.27 (0.03)	0.22	0.24 (0.03)	0.25	0.24 (0.03)	0.26	0.24 (0.02)	0.24 (0.02)	0.25 (0.03)
≥65	0.10	0.14 (0.06)	0.12	0.11 (0.03)	0.12	0.10 (0.02)	0.14	0.11 (0.02)	0.12 (0.02)	0.12 (0.04)
Race and ethnicity										
Black	0.05	0.03 (0.02)	0.09	0.06 (0.06)	0.26	0.12 (0.04)	0.05	0.05 (0.03)	0.11 (0.10)	0.07 (0.05)
Hispanic	0.12	0.15 (0.07)	0.11	0.25 (0.10)	0.26	0.43 (0.17)	0.15	0.36 (0.18)	0.16 (0.07)	0.30 (0.17)
White	0.49	0.52 (0.11)	0.56	0.39 (0.08)	0.27	0.24 (0.09)	0.41	0.34 (0.13)	0.43 (0.12)	0.37 (0.14)
Households below poverty	0.11	0.07 (0.03)	0.19	0.13 (0.06)	0.20	0.16 (0.05)	0.13	0.15 (0.04)	0.16 (0.05)	0.13 (0.06)
Education										
High school or less	0.10	0.19 (0.05)	0.13	0.24 (0.09)	0.36	0.46 (0.09)	0.25	0.35 (0.11)	0.21 (0.12)	0.31 (0.14)
Some college	0.17	0.25 (0.02)	0.18	0.25 (0.04)	0.25	0.32 (0.03)	0.20	0.28 (0.05)	0.20 (0.04)	0.27 (0.05)
College degree or higher	0.73	0.56 (0.06)	0.70	0.51 (0.13)	0.39	0.22 (0.08)	0.54	0.37 (0.15)	0.59 (0.16)	0.42 (0.17)

^a^ Data are averages of 4 years of American Community Survey 5-year averages starting with 4 years before exposure year (example exposure year is 2017, and value is average of American Community Survey 5-year averages for 4 years starting with 2013).

^b^ Data are mean values for exposure cities.

^c^ Data are mean (SD).

The main analysis included 390 199 patients with approximately 2.2 million person-year records. A total of 44 771 youth aged 2 to 19 years (mean [SD] age, 6.4 [4.2] years; 22 337 female [49.9%]) resided in the exposure cities; 345 428 youth (mean [SD] age, 6.9 [4.2] years; 171 018 female [49.5%]) resided in control cities, as displayed in Table 2. Although the mean age and the percentage of female individuals were similar across exposure and control cities, there were demographic differences. Of note, the race and ethnicity distribution and percentage enrolled in state-subsidized insurance plans among KP patients in exposure cities were not comparable in some cases. Hispanic youth were overrepresented in the control cities for Berkeley, San Francisco, and Oakland, whereas Black youth were underrepresented in the control cities for Oakland and Berkeley. However, the age-matched and sex-matched BMI percentiles, as well as patients classified with overweight or obesity, was similar in exposure and control cities. Across all 44 cities, approximately one-quarter of KP youth patients met the definition for overweight or obesity.

**Table 2.** Characteristics of Exposure and Control Cities for Kaiser Permanente Members Aged 2 to 19 Years at Baseline (First Year of Cohort Entry)

Characteristic	Participants, No. (%)									
	Albany		Berkeley		Oakland		San Francisco		Total^a^	
	Exposure (n = 1263)	Controls (n = 30 938)	Exposure (n = 3744)	Controls (n = 23 500)	Exposure (n = 18 981)	Controls (N = 122 106)	Exposure (n = 20 826)	Controls (n = 169 509)	Exposure (N = 44 771)	Controls (N = 345 428)
Age, mean (SD), y	7.3 (4.1)	7.1 (4.2)	6.7 (4.2)	6.9 (4.3)	6.4 (4.2)	7.0 (4.2)	6.4 (4.2)	6.9 (4.2)	6.4 (4.2)	6.9 (4.2)
Race and ethnicity										
Asian	355 (28.1)	10 566 (34.2)	335 (9.0)	5476 (23.3)	2217 (11.7)	19 461 (15.9)	6579 (31.6)	36 515 (21.5)	9481 (21.2)	71 847 (20.8)
Black	41 (3.3)	732 (2.4)	418 (11.2)	817 (3.2)	4610 (24.3)	16 194 (13.3)	1260 (6.1)	8574 (5.1)	6324 (14.1)	26 247 (7.6)
Hispanic	130 (10.13)	4288 (13.9)	494 (13.2)	7870 (33.5)	5611 (29.6)	54 003 (44.2)	3856 (18.5)	71 380 (42.1)	10 077 (22.5)	137 306 (39.8)
White	537 (42.5)	11 696 (37.0)	2031 (54.3)	7384 (31.4)	4214 (22.2)	22 466 (18.4)	5529 (26.6)	40 944 (24.2)	12 299 (27.5)	82 373 (23.9)
Other^b^	200 (15.8)	3656 (11.8)	466 (12.5)	1953 (8.3)	2329 (12.3)	9982 (8.2)	3602 (17.3)	12 096 (7.1)	6590 (14.7)	27 655 (8.0)
Sex										
Female	639 (50.6)	15 348 (49.6)	1832 (48.9)	11 505 (49.0)	9548 (50.3)	60 762 (49.8)	10 341 (49.7)	83 713 (49.4)	22 337 (49.9)	171 018 (49.5)
Male	624 (49.4)	15 590 (50.4)	1912 (51.1)	11 995 (51.0)	9433 (49.7)	61 344 (50.2)	10 485 (50.4)	85 796 (50.6)	22 434 (50.1)	174 410 (50.5)
State-subsidized insurance plan	58 (4.6)	1471 (4.8)	148 (4.0)	3125 (13.3)	2443 (12.9)	26 974 (22.1)	1786 (8.6)	26 576 (15.7)	4431 (9.9)	58 007 (16.8)
Neighborhood household poverty >20%^c^	57 (4.7)	185 (0.6)	570 (16.1)	1181 (5.7)	7398 (42.3)	22 134 (19.7)	1171 (6.3)	21 150 (14.1)	9191 (22.5)	44 605 (14.3)
Body mass index percentile, mean (SD)^d^	52.2 (28.2)	53.5 (29.9)	56.0 (28.2)	57.8 (30.3)	60.3 (29.5)	61.1 (30.7)	57.19 (29.1)	58.73 (30.7)	58.3 (29.3)	59.0 (30.7)
Youth with overweight or obesity^e^	210 (16.6)	6233 (20.2)	703 (18.8)	6006 (25.6)	5279 (27.8)	37 830 (31.0)	4788 (23.0)	47 198 (27.8)	10 969 (24.5)	97 061 (28.1)
Youth with overweight (not obesity)^f^	149 (11.8)	3868 (12.5)	433 (11.6)	3036 (12.9)	2606 (13.7)	17 929 (14.7)	2725 (13.1)	23 182 (13.7)	5906 (13.2)	47 904 (13.9)
Youth with obesity^g^	61 (4.8)	2365 (7.6)	270 (7.2)	2970 (12.6)	2673 (14.1)	19 901 (16.3)	2063 (9.9)	24 016 (14.2)	5063 (11.3)	49 157 (14.2)

^a^ The total exposure and total controls do not add up to these totals. There were 625 controls and 43 exposure patients who qualified to be included in 2 separate analyses and were only included only 1 time in the total count.

^b^ Other race refers to American Indian or Alaska Native, multiracial, and other race and ethnicities not listed or unknown.

^c^ Neighborhood is defined as the US Census tract of a patient’s residence.

^d^ Body mass index is calculated as weight in kilograms divided by height in meters squared.

^e^ Refers to body mass index percentile greater than 85th percentile.

^f^ Refers to body mass index percentile greater than 85th percentile to less than or equal to 95th percentile.

^g^ Refers to body mass index percentile greater than 95th percentile.

The Figure displays the unadjusted mean BMI percentile trajectories over time in all cities during the pretax and posttax periods. Although BMI percentile was consistently lower in exposure compared with control cities, an upward trend in the control cities of Berkeley and San Francisco was noted in the posttax period.

**Figure.**
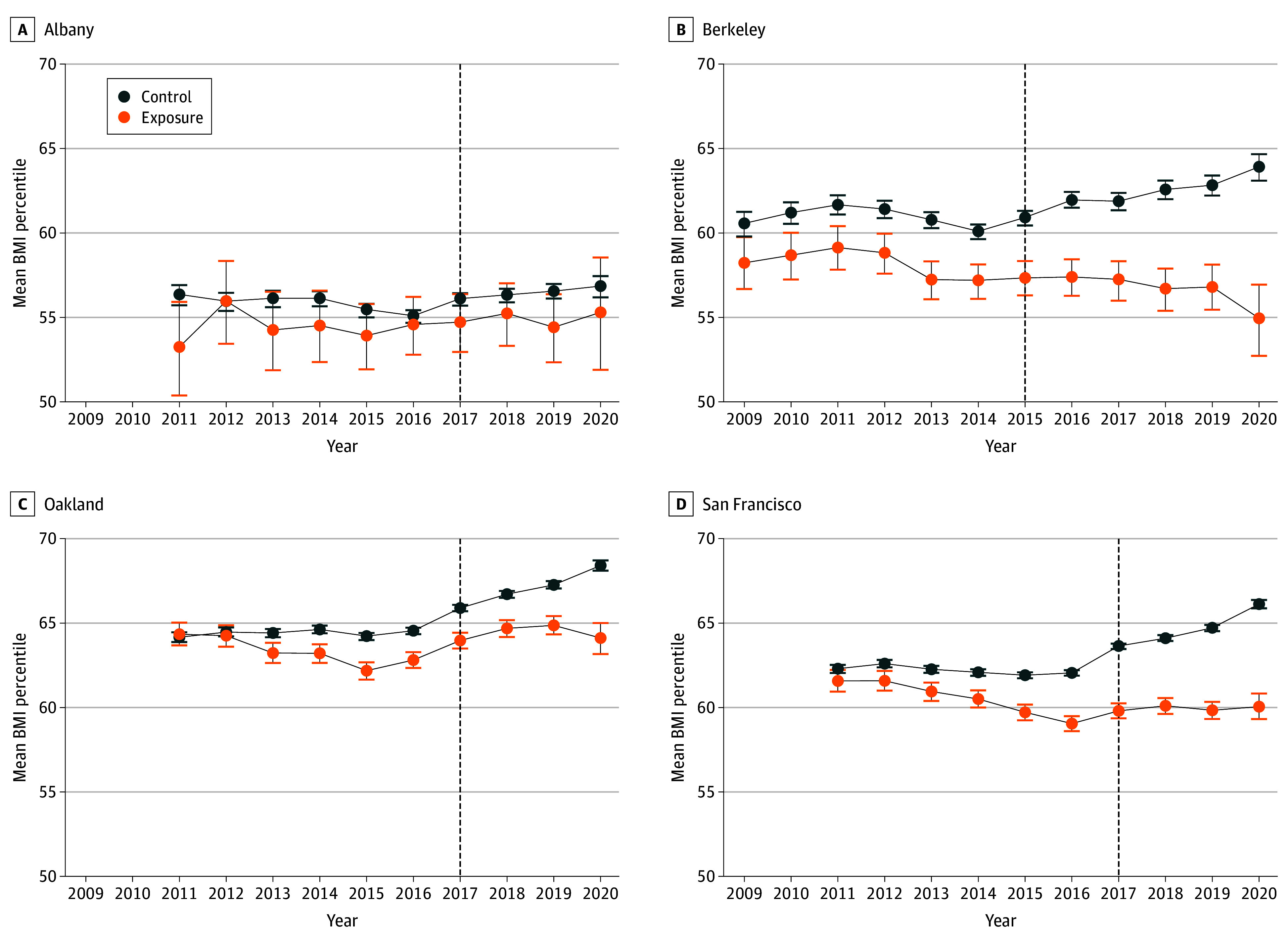
Body Mass Index (BMI) Percentile of Youth Aged 2 to 19 Years in 4 California Cities Graphs show before and after trends in mean BMI (weight in kilograms divided by height in meters squared) percentiles from 2009 to 2020 in each city in California that implemented sugar-sweetened beverage taxes compared with the mean of each city’s 10 control cities. Error bars denote SDs.

All block level models passed the placebo test except for 1 small block for the BMI percentile outcome. Table 3 displays aggregated results for the overall and subgroup effect estimates from the DID analysis. Across the 4 exposure cities, the mean BMI percentile decreased significantly before and after the SSB tax implementation vs changes in the control cities (−1.64 percentage points; 95% CI, −3.10 to −0.17 percentage points). However, there were no significant differences in the changes of the percentage of youth who were classified with overweight or obesity (−2.01 percentage points; 95% CI, −4.58 to 0.56 percentage points) or with obesity (−1.15 percentage points; 95% CI, −2.76 to 0.45 percentage points) between the exposure and control cities.

**Table 3.** Overall and Subgroup Results by the Differences-in-Difference Analysis^a^

Variable	Percentage points (95% CI)		
	Additive differences in the mean BMI percentile^b^	Youth with overweight or obesity	Youth with obesity
Overall outcome	−1.64 (−3.10 to −0.17)	−2.01 (−4.58 to 0.56)	−1.15 (−2.76 to 0.45)
Subgroup outcomes			
Tax city			
Albany	−0.16 (−3.16 to 2.84)	−0.66 (−6.19 to 4.87)	−0.93 (−4.97 to 3.11)
Berkeley	−3.55 (−5.20 to −1.90)	−4.44 (−7.81 to −1.07)	−1.60 (−4.49 to 1.30)
Oakland	−1.00 (−2.43 to 0.43)	−1.02 (−3.60 to 1.56)	−1.20 (−2.93 to 0.53)
San Francisco	−1.97 (−3.34 to −0.60)	−2.56 (−4.79 to −0.33)	−1.05 (−2.15 to 0.06)
Age range in 2017, y			
2-5	−2.06 (−4.04 to −0.09)	−5.46 (−8.47 to −2.44)	−1.87 (−3.36 to −0.38)
6-11	−2.79 (−4.29 to −1.30)	−4.23 (−6.90 to −1.57)	−1.85 (−3.46 to −0.24)
12-19	−1.14 (−2.45 to 0.18)	−0.78 (−4.25 to 2.70)	−0.16 (−1.91 to 1.59)
Sex			
Female	−1.29 (−2.72 to 0.14)	−1.5 (−4.08 to 1.08)	−0.72 (−2.15 to 0.72)
Male	−1.98 (−3.48 to −0.48)	−2.52 (−5.08 to 0.03)	−1.59 (−3.36 to 0.18)
Race and ethnicity			
Asian	−1.63 (−3.10 to −0.16)	−2.10 (−4.89 to 0.53)	−0.68 (−1.90 to 0.57)
Black	−0.82 (−2.53 to 0.88)	−0.02 (−3.42 to 3.38)	−0.46 (−2.75 to 1.82)
Hispanic	−1.22 (−2.47 to 0.03)	−1.12 (−3.39 to 1.15)	−0.49 (−2.19 to 1.21)
White	−2.58 (−4.11 to −1.10)	−3.73 (−6.11 to −1.35)	−2.78 (−4.18 to −1.37)
Other^c^	−1.30 (−2.72 to 0.13)	−1.80 (−4.08 to 0.48)	−0.47 (−2.14 to 1.20)
Year^d^			
Year 1	−1.13 (−3.02 to 0.77)	−1.14 (−4.45 to 2.17)	−0.78 (−2.90 to 1.35)
Year 2	−1.22 (−3.22 to 0.77)	−1.66 (−5.17 to 1.86)	−1.06 (−3.31 to 1.19)
Year 3	−1.63 (−3.74 to 0.49)	−2.03 (−5.78 to 1.72)	−1.05 (−3.44 to 1.34)
Year 4	−2.26 (−5.24 to 0.72)	−2.85 (−8.32 to 2.63)	−1.75 (−5.14 to 1.65)
Youth with overweight or obesity^e^	−0.71 (−1.48 to 0.06)	NA	NA
Youth with obesity^e^	−0.58 (−0.90 to −0.26)	NA	NA
High poverty cohort^f^	−0.52 (−2.22 to 1.18)	NA	NA

Abbreviations: BMI, body mass index; NA, not applicable.

^a^ Analysis is jointly controlled for race and ethnicity, age, sex, insurance status, and city by year fixed effects, as well as intraclass correlation within patients.

^b^ BMI is calculated as weight in kilograms divided by height in meters squared.

^c^ Other race refers to American Indian or Alaska Native, multiracial, and other race and ethnicities not listed or unknown.

^d^ Year 1 is the tax year. For Berkeley, this is 2015, for the remaining cities it is 2017.

^e^ Overweight or obesity subgroup was BMI percentile greater than 85th at baseline (first year of cohort entry), and obesity subgroup was BMI percentile greater than 95th at baseline.

^f^ Refers to primary residence with neighborhood household poverty greater than 20% based on US Census data.

There were significant outcomes in some population subgroups (Table 3). In youth with obesity at cohort entry, the change in the mean BMI percentile before and after the tax implementation was significant in exposure cities compared with control cities (−0.58 percentage points; 95% CI, −0.90 to −0.26 percentage points). After SSB tax implementation, there were statistically significant outcomes in the cities of Berkeley and San Francisco. Compared with the temporal trends of their control cities, both Berkeley (−3.55 percentage points; 95% CI, −5.20 to −1.90 percentage points) and San Francisco (−1.97 percentage points; 95% CI, −3.34 to −0.60 percentage points) had significant reductions in the mean BMI percentile. Berkeley (−4.44 percentage points; 95% CI, −7.81 to −1.07 percentage points) and San Francisco (−2.56 percentage points; 95% CI, −4.79 to −0.33 percentage points) also had significant reductions in the percentages of youth classified with overweight or obesity compared with changes in control cities. Compared with changes in control cities, younger youth residing in the exposure cities had significant reductions in mean BMI percentile, including youth aged 2 to 5 years in 2017 (−2.06 percentage points; 95% CI, −4.04 to −0.09 percentage points) and youth aged 6 to 11 years in 2017 (−2.79 percentage points; 95% CI, −4.29 to −1.30 percentage points). The association was also significant for younger youth in the percentages of overweight or obesity (ages 2-5 years, −5.46 percentage points; 95% CI, −8.47 to −2.44 percentage points; ages 6-11 years, −4.23 percentage points; 95% CI, −6.90 to −1.57 percentage points) and the percentages of children with obesity (ages 2-5 years, −1.87 percentage points; 95% CI, −3.36 to −0.38 percentage points; ages 6-11 years, −1.85 percentage points; 95% CI, −3.46 to −0.24 percentage points). Male youth in exposure cities had significant changes in BMI percentile compared with those in the control cities (−1.98 percentage points; 95% CI, −3.48 to −0.48 percentage points). Significant reductions in mean BMI percentile after SSB tax implementation vs changes in control cities were noted for Asian youth (−1.63 percentage points; 95% CI, −3.10 to −0.16 percentage points) and White youth (−2.58 percentage points; 95% CI, −4.11 to −1.10 percentage points). Estimates among Hispanic youth were also notable but not statistically significant (−1.22 percentage points; 95% CI, −2.47 to 0.00 percentage points; *P* = .06). White youth also had improvements in the percentage overweight or obese (−3.73 percentage points; 95% CI, −6.11 to −1.35 percentage points) and the percentage with obesity (−2.78 percentage points; 95% CI, −4.18 to −1.37 percentage points). Compared with trends in control cities, no significant changes in the mean BMI percentile were found among youth enrolled in state-subsidized insurance plans (data not shown), and no significant associations were found in the high-poverty subcohort (−0.52 percentage points; 95% CI, −2.22 to 1.18 percentage points). Estimates from the sensitivity analyses mirrored those of the main analyses, but the findings were not statistically significant (eg, for the overall association with BMI percentile) owing to the SCM’s lack of power (eAppendix, eFigure, and eTable 2 in Supplement 1).

## Discussion

This cohort study found that after implementation of SSB excise taxes, youth living in exposure cities with SSB taxes had significantly lower BMI percentile compared with youth living in control cities. Statistically significant associations were noted for youth younger than 12 years, male individuals, White and Asian youth, and youth with obesity, and were particularly high in the cities of Berkeley and San Francisco. Improvements were noted in lower prevalence of overweight or obesity and obesity in some population subgroups. Although the associations were modest, they were consistent across cities and population subgroups. Sensitivity analyses offered support to the robustness of the results in the main analyses, although the sensitivity analyses did not achieve statistical significance. Our results demonstrate that SSB excise taxes may be associated with lower BMI percentile and lower prevalence of obesity among youth. Policymakers and public health leaders should consider implementing taxes on SSBs as a lever to reduce BMI percentile among our nation’s youth and reduce overweight and obesity for children younger than 12 years.

Others have estimated the potential health outcomes of applying SSB excise taxes to decrease consumption. Those estimates suggested greater outcomes for adolescents.^16^ Flynn^18,19^ noted reductions in self-reported soda consumption among high school students in Philadelphia after SSB taxes were implemented and reductions in self-reported BMI among youth in Philadelphia, Oakland, and San Francisco compared with control cities using Youth Risk Behavioral Surveillance System data. His study design, however, differed from ours. We found that over 3 to 5 years after tax implementation, children aged 2 to 5 years had a 5.46–percentage point reduction in overweight or obesity and a 1.87–percentage point reduction in obesity prevalence compared with control cities and that children aged 6 to 11 years had a 4.23–percentage point reduction in percentage of overweight or obesity compared with control cities. Our results suggest that associations of the taxes with youth weight status may be greater among younger children.

Previous studies^29,33,34,35^ suggested that SSB taxes would result in the greatest health gains for people with lower incomes and underrepresented racial and ethnic groups.^29^ Four months after the Berkeley SSB excise tax was implemented, consumption declined by 21% in low-income neighborhoods.^12^ After SSB excise taxes were implemented in San Francisco, Silver and colleagues^14^ noted a reduction in being classified as a high SSB consumer compared with the control city (San Jose, California) among low income consumers. Those studies assessed consumption in adults, however. Although the present study did not assess whether or how the SSB tax affected actual SSB intake among youth, we did not observe statistically significant weight-related benefits among low income or Black youth; effect estimates were attenuated but in the same direction as in other groups. Further studies are needed to investigate why the outcomes of the tax may have been attenuated in some subgroups, despite evidence suggesting an impact on SSB consumption, and whether this is the case in other US jurisdictions with SSB excise taxes. Perhaps including data on neighborhood environments, which are associated with BMI trajectories and obesity risk,^36^ could be investigated in future studies. Living in poor-resourced environments may outweigh the possible beneficial outcomes of SSB taxes.

Revenues generated from SSB taxes have been used, in part, for obesity prevention initiatives.^37,38^ Krieger et al^15^ described how revenue from the SSB taxes in US cities with these taxes were allocated up to the year 2021. Annual revenues ranged from $273 000 in Albany to $16 098 000 in San Francisco, with most of the revenues supporting health-related goals (100% in Albany, 92% in Berkeley, 79% in San Francisco, and 51% in Oakland). Although the revenues were used for many purposes, youth were a priority population in all SSB tax cities, with 73% of taxes in Berkeley, 52% in Albany, 46% in San Francisco, and 32% in Oakland focused on youth.^15^ Although we cannot speculate whether reductions in purchasing and consumption or increased health-related programming for youth were responsible for the results we found, the results suggest that implementing excise taxes on SSB distributors improved weight-related outcomes for youth.

### Limitations

There are limitations to our work. The cohort consisted of KP members, who may have different characteristics than other residents of the study cities. The DID approach required that cohort members have at least 1 BMI measurement before and after implementation, which also restricts generalizability. Additionally, the 4 exposure cities have demographics slightly different than those for most of California, and our candidate pool for control cities was limited to California cities where KP members reside; however, our matching algorithm was able to create matches using the best available cities. Nonetheless, Black youth were more prevalent in Berkeley and Oakland than in their control cities. Our methods cannot discern whether outcomes after SSB tax implementation were through reduction in consumption, additional programming from the tax revenues, or other potential causal pathways. We acknowledge that there are no relevant mediators available in the KP EHR system related to SSB consumption. Future studies can address mediators of tax outcomes on SSB intake.

## Conclusions

In this cohort study of the outcomes of city-level SSB excise tax implementation, the policy may have prevented increases in BMI percentile among youth. These findings support the importance of excise taxes in influencing purchases and consumption of beverages known to have a negative impact on health. Policymakers and public health leaders should strongly consider implementing excise taxes on SSBs as a lever to curtail the increase in BMI percentile among youth and overweight and obesity among youth younger than 12 years.

## Notes

Supplement 1. **eAppendix.** Technical details of the statistical approaches **eTable 1.** List of the 44 study cities
eReferences
**eFigure.** Average outcome in each study year for each tax city and corresponding synthetic control **eTable 2.** Overall and subgroup intervention effects using the synthetic control method, aggregated over the 4 intervention cities with permutation-based p-values

Supplement 2.
Data Sharing Statement
