# Supplementary material for: City-Level Sugar-Sweetened Beverage Taxes and Youth Body Mass Index Percentile
Source: JAMA Netw Open. 2024 Jul 31;7(7):e2424822. doi: 10.1001/jamanetworkopen.2024.24822 (PMC11292449; doi:10.1001/jamanetworkopen.2024.24822)
Supplement: Supplement 1. — eAppendix. Technical details of the statistical approaches eTable 1. List of the 44 study cities eReferences eFigure. Average outcome in each study year for each tax city and corresponding synthetic control eTable 2. Overall and subgroup intervention effects using the synthetic control method, aggregated over the 4 intervention cities with permutation-based p-values [file jamanetwopen-e2424822-s001.pdf]

# Supplemental Online Content

Young DR, Hedderson MM, Sidell MA, et al. City-level sugar-sweetened beverage taxes and youth body mass index percentile. *JAMA Netw Open*. 2024;7(7):e2424822. doi:10.1001/jamanetworkopen.2024.24822

**eAppendix.** Technical details of the statistical approaches

**eTable 1.** List of the 44 study cities

**eReferences**

**eFigure.** Average outcome in each study year for each tax city and corresponding synthetic control

**eTable 2.** Overall and subgroup intervention effects using the synthetic control method, aggregated over the 4 intervention cities with permutation-based p-values

This supplemental material has been provided by the authors to give readers additional information about their work.

© 2024 Young DR et al. *JAMA Network Open.*

**eAppendix.** Technical details of the statistical approaches

## 1. The study city matching procedure

Data used for matching

The California sugar sweetened beverage (SSB) Tax Study is a policy evaluation study using a natural experiment design. The intervention arm consisted of four California cities that adopted the SSB excise tax between 2015 and 2017 (Albany, Berkeley, San Francisco, and Oakland). The four intervention cities are all located in the greater San Francisco Bay area with distinct city-level characteristics. Potential control cities included 328 incorporated cities in California, where health care is covered by Kaiser Permanente (KP) and no SSB excise tax was in effect between 2009 and 2020. Seventeen city-level covariates were collected: total population, population density , % population having KP membership, % males, % females, % population in each of the four age strata (≤ 19, 20-44, 45-64, ≥ 65), % population in each of the four race/ethnicity categories (Hispanic of all races, non-Hispanic African American, non-Hispanic white, non-Hispanic Asian and others), % population living below poverty line, and % population in each of the three education attainment levels (high school diploma/GED or lower, some college or associate degree, bachelor degree or higher). Except for % population having KP membership, all other covariates are public information using 5-year averages of U.S. Census American Community Survey prior to SSB tax implementation years.

Matching

Weighted Euclidean distance metrics were calculated using 14 of the 17 covariates by removing a redundant category from three factors (% males, ≥ 65 years old, bachelor's degree or higher) and after standardization (i.e., dividing the raw value by the population standard deviation) using the formula

$$d(p,q) = \sqrt{\sum_{i=1}^{14} w_i (q_i - p_i)^2}$$

where $p=(p_1, p_2, \ldots, p_{14})$, $q=(q_1, q_2, \ldots, q_{14})$ are two points in the 14-dimension Euclidean space, $q_i$, $p_i$, $i=1, \ldots, 14$ are standardized matching dimensions, and $w_i$ = weight for dimension i.

Weights were assigned to down weight percentages in the same factor (e.g., the three percentages for age strata each had a weight of .333) so that the total weight of a factor with more than one level was 1. All pairwise distance metrics between a treatment city and a potential control city were calculated. Each intervention city was matched to 10 control cities with generally the shortest distance metrics. Minor ad hoc adjustments were made to avoid overlap in selected control cities among different intervention cities and to ensure no control cities bordered any intervention cities. Specifically, first we excluded any matched city that was geographically adjacent to the intervention city and replaced it with the next available control city from the list ordered by Euclidean distance. Next, we examined controls to ensure that each control city was only matched to one intervention city. If a city was matched to multiple intervention cities, the match with the smallest corresponding Euclidean distance was maintained while the other was replaced with that intervention city's next available control city from the list ordered by Euclidean distance. The final list of intervention and matched control cities is listed in **eTable 1.**

Balance assessment

Balance assessment was ascertained by examining the absolute standardized difference between the mean value (SMD) for matched control cities and the value of each covariate in the intervention city. Due to the small sample size and the finite study population of California cities, some covariates have moderately large SMD. We conducted a Monte Carlo simulation to assess the relative goodness of balance. Specifically, 100,000 ideal random samples of California cities were drawn (4 treated cities and 40 control cities in each random sample). The SMD of our chosen study sample is smaller than or close to the median SMD of the 100,000 ideal random samples. Details of the simulations are reported elsewhere. (Han and Sidell, 2024) Therefore, we concluded that in relation to an ideal simple randomization, the chosen study sample had acceptable levels of covariate balance.

© 2024 Young DR et al. *JAMA Network Open.*

**eTable 1. List of the 44 study cities.**

| Tax City | Matched Comparison Cities |
|---|---|
| Albany | San Ramon, Dublin, Pleasanton, Santa Clara, San Rafael, Burlingame, Walnut Creek, Belmont, Claremont, Lafayette |
| Berkeley | Davis, Pasadena, Loma Linda, Sunnyvale, Fullerton, San Mateo, Tustin, Signal Hill, South Pasadena, Placentia |
| Oakland | Sacramento, Moreno Valley, Pittsburg, Stockton, Elk Grove, Rialto, Hayward, Fontana, San Leandro, Corona |
| San Francisco | San Jose, San Diego, Irvine, Anaheim, Fremont, Riverside, Long Beach, Chula Vista, Glendale, Bakersfield |

## 2. The stratified difference-in-differences (DID) method

Overview

The difference-in-differences (DID) method is a widely applied causal inference method, conventionally operationalized through an Analysis of Covariance (ANCOVA) longitudinal regression model. The key assumption of the DID method was that in the absence of any treatment, the trajectories of the mean outcome would have been parallel between any study arms (Lechner, 2011). We adopted the DID method as the main analytic approach in this paper. However, the massive study sample from KP's electronic health record (EHR) imposed a great challenge. Standard ANCOVA longitudinal regression models, including the many variants, could not pass the general falsification and validation tests for model specification and the fundamental parallel trajectory assumption. The standard ANCOVA regression might yield biased estimates for the SSB tax treatment effects as well as potentially false significance. To ensure unbiased estimates and avoid type I errors, we first stratified the full sample into independent subsamples of blocks. In each block, observed covariates were completely homogenous or almost so. The sample size in each block was reasonably small. Thus, adjusting for covariates was either not needed or could be adequately done within each block. Second, the trajectories of the mean outcome were fully nonlinear in each block. These two adjustments made the analysis in each block adequately verified. Finally, results from all blocks were aggregated to form the overall estimates and subgroup estimates. Details of these steps were as follows.

Data, conventional DID, and validation and falsification tests

We first introduced the standard notation for unsynchronized longitudinal data: the outcome data were denoted as $Y(i,t_{i,j})$ for patient $i=1,\ldots,N$, $N\approx3.9\times10^5$ and in year $t_{i,j}$. Patient i had repeated measurements in a sequence of years $\{t_{ij}: t_{i,1}, \ldots, t_{i,Ti}\}$. All years $t_{i,j}$ ranged between 1 and 10, where the first six years were pre-tax and the last four years were post-tax (ignoring the different timeline for Berkely for the sake of brevity). We required that the number of measurements in patient i be $Ti \geq 2$, the first measurement time $t_{i,1}<7$, and the last measurement time $t_{i,Ti} \geq 7$. Except for these requirements, all patients could have different numbers of measurement and different measurement times. The total number of measurements was $\sum_{i=1}^{N} Ti \approx 2.2\times10^6$. Let $Z_i$ = 1 or 0 denote whether a patient in a treated city, and $X_i$ denote the observed baseline covariate vector (birth year, gender, race/ethnicity, insurance status, and specific city of residence). Let I{condition} denote a dummy variable for the condition within the brackets. With this notation system, a basic ANCOVA model had the following mean function

$$E[Y(i,t_{i,j})] = \alpha_1 + Z_i\alpha_2 + \gamma t + \mu t Z_i I\{t>6\} + X_i\beta, \quad (1)$$

where the parameter of interest was μ (i.e., the coefficient for the time by condition interaction). Other parameters in the mean function include the intercept $\alpha_1$ for mathematical purposes, the baseline difference between arms $Z_i\alpha_2$, the adjusted covariate effect $X_i\beta$, and the common trajectory $\gamma t$, which would be followed by everyone in the absence of treatment. The variance components of the ANCOVA model besides Equation (1) included the random measurement error, the serial correlation within a patient, and potentially the clustering within a city, where the last two error components could be modeled by random effects, covariance terms, or the fixed-effect approach. Each approach further had many technical variants. In the sequel, we focused on the mean function and omitted most tedious technical details in variance components.

The main challenge for the DID approach was encountered in the two types of validation and falsification tests. First, a goodness-of-fit test checked if the working model underfitted the data. Due to the massive sample size and highly heterogeneous patients (390k patients, 2.2 million records), it was almost certain that a conventional

© 2024 Young DR et al. *JAMA Network Open.*

parametric regression could not adequately fit the data. Minor enrichments of model (1), such as short-term shock, two-way interactions, parabolic or cubic time trends, or regression spline in time trends, were of little to no help. For example, a slightly enriched model for (1) was to replace the parameter of interest $\mu t Z_i I\{t>6\}$ by two terms $\mu_1 Z_i I\{t>6\} + \mu_2 t Z_i I\{t>6\}$, i.e., an instant shock and a long-term linear effect. Not surprisingly, the enriched model had significantly better goodness of fit than the working model by any reasonable goodness-of-fit test or model selection/comparison criteria. However, the enriched model itself also substantially underfitted the data and could use further expansion.

Sometimes a model's goodness-of-fit was deemed as a technical concern rather than a substantive jeopardy. However, the placebo test for DID was always deemed as crucial to partially justify its validity. The rationale of the placebo test was to conduct the DID analysis in the pre-tax period only by setting a fake and shortened post-tax period, e.g., using years 1 to 5 as pre-tax and year 6 as the fake post-tax period. Since no treatment was in effect, the placebo test was expected to be insignificant if the parallel trajectory assumption was not violated. Nevertheless, it was almost certain that any conventional parametric regression to DID could not pass the placebo test. Lastly, the massive dataset also made it challenging to fit most conventional models and perform these tests computationally: a single mixed-effect model could take more than several days to fit in SAS Studio Enterprise 9.4.

Our stratified and saturated DID

Stratification: as recommended by the official user manual of SAS/STAT software, we partitioned the sample into mutually exclusive subsamples or blocks to reduce the total computational burden, where blocks were defined by intervention city, race and ethnicity, sex, birth years, and insurance status jointly. For example, a block was Hispanics, males, having Medicaid or other public health insurance, born between 2005 and 2006, living in San Francisco or its 10 matched control cities. Another block was white, females, any insurance, born between 2006 and 2010, living in Oakland or its 10 matched control cities. In the end, we used 104 blocks for the analysis of the child BMI percentile outcome. Each block had a reasonably small sample size, usually between 10,000 to 100,000 records. Moreover, the observed covariates were either constant or had minor differences within each block so that goodness-of-fit for covariates was no longer a concern.

Saturated DID: within each block there was still the daunting task to adequately model the mean trajectories to pass the placebo test. We employed a fully nonlinear and saturated parametrization based on the robust DID approach in the econometric literature (Conley and Taber, 2011; Rambachan and Roth, 2023). Let $Y^{(g)}_{(i,k,j)}$ denote the outcome for patient i in city k at time j, $1 \leq j \leq 10$, k=1 for the treated city and k=2,…11 for control cities, and the superscript (g) denoted a distinct block independent of all other blocks. Note that all blocks had exactly 11 cities as presented here. The saturated DID for block g is

$$E[Y^{(g)}_{(i,k,j)}] = \lambda^{(g)}_{k,j} + X_i^{*(g)}\beta^{(g)}. \qquad (2)$$

where each city had a fully flexible trajectory $\lambda^{(g)}_{k,1}, \lambda^{(g)}_{k,2}, \ldots, \lambda^{(g)}_{k,10}$, and the term $X_i^{*(g)}\beta^{(g)}$ adjusted for the few remaining non-constant covariates. Statistically, the 110 distinct parameters $\lambda^{(g)}_{k,j}$ in block g were the highest-order interaction term that could be applied to the mean function. All variance components were modeled separately for each block as well. Thus, the collection of all block-level models composed a very large-scale parametrization with roughly 110 x 104 ≈ $1.1 \times 10^4$ parameters, resulting in a "n-p ratio" of roughly 190, i.e., 190 data points per unknown parameter for estimation. (Note: the actual number of parameters is slightly more than this due to the variance components and the few covariate terms). We used the following placebo test for model (2)

$$H_0: \left[\lambda^{(g)}_{1,6} - \tfrac{1}{5}\textstyle\sum_{j=1}^{5}\lambda^{(g)}_{1,j}\right] - \left[\textstyle\sum_{k=2}^{11} v_k^{(g)}\lambda^{(g)}_{k,6} - \tfrac{1}{5}\textstyle\sum_{j=1}^{5}\sum_{k=2}^{11} v_k^{(g)}\lambda^{(g)}_{k,j}\right] = 0,$$

where control cities had weights $v_k^{(g)}$, k=2, …,11. These weights were equal to 0.1 by default (i.e., 10 controls equally weighted). Causal effect contrasts were estimated only if the placebo test could pass, i.e. fail to reject $H_0$. In the event that the placebo test failed to pass, we made one or more of the following adjustments, including splitting the block, combining the block with an adjacent block, or combining and re-splitting the block with adjacent block(s). Failure to pass the placebo test might also result from one or more control cities whose trajectories were substantially different from other control cities and the treated city. In these cases, we set $v_k^{(g)} = 0$ to exclude these control cities and adjusted the weights or the remaining control cities in this block. For example, if we decided to exclude one control city in a block, then this excluded city's weight was 0 and the other 9 control cities weight was changed to 0.1111.

After passing the placebo test, the causal effect for a post-tax year t, $10 \geq t \geq 7$, was the following linear contrast

$$L_t^{(g)} = \left[\lambda^{(g)}_{1,t} - \tfrac{1}{6}\textstyle\sum_{j=1}^{6}\lambda^{(g)}_{1,j}\right] - \left[\textstyle\sum_{k=2}^{11} v_k^{(g)}\lambda^{(g)}_{k,t} - \tfrac{1}{6}\textstyle\sum_{j=1}^{6}\sum_{k=2}^{11} v_k^{(g)}\lambda^{(g)}_{k,j}\right].$$

© 2024 Young DR et al. *JAMA Network Open.*

The causal effect for the overall effect across four post-tax years was the following linear contrast

$$L^{(g)} = \frac{1}{4}\sum_{t=7}^{10} L_t^{(g)}.$$

All causal effect contrasts and the placebo test statistics were estimable under the general linear hypothesis inference framework (McLean et al., 1991).

Aggregation operation

Aggregated point estimates and standard errors were calculated by taking the overall mean of the block estimates weighted by proportion of distinct subjects in the intervention city $w^{(g)}$,

$$L_t = \sum_g w^{(g)} L_t^{(g)}, \text{ and } L = \sum_g w^{(g)} L^{(g)}.$$

Subgroup (race/ethnicity, age, and sex) point estimates were calculated using the same method above but with the summation over blocks sharing the common subgroup characteristics and weights standardized to sum to one among these blocks. By statistical independence among blocks, the SE of the contrasts $L_t$, L, and subgroup effects were square roots of the sum of squared SEs from all blocks involved. We applied the Wald's z test inference to calculate 95% confidence intervals and p-values for the aggregated results.

The full technical details and software codes of the stratified saturated DID approach will be reported elsewhere and are available upon requests. All data analysis by the DID approach was conducted using PROC MIXED in SAS Studio Enterprise version 9.4 (SAS Institute Inc., Cary, NC, USA).

### 3. The synthetic control approach

The synthetic control approach served as sensitivity checks to verify the substantive findings by the DID approach. Compared with the DID approach, the synthetic control approach used slightly different input data, imposed distinct causal inference assumptions, and used fundamentally different estimators.

Data used in the synthetic control analysis

The synthetic control method analyzes aggregated data at the city-level. All available patient-level outcomes from the EHR, minus the invalid records as described in the method section, were summarized by city and calendar year using means. We did not require more than one repeated measure in the sensitivity analysis by the synthetic control method. The analytic data for the synthetic control method included mean outcomes from 44 cities (4 intervention and 10 controls for each intervention city). The average annual number of youth in each intervention city and the corresponding matched control cities are displayed below.

| | **Albany** | **Berkeley** | **Oakland** | **San Francisco** |
|---|---|---|---|---|
| Intervention city | 1,148 | 3,365 | 16,876 | 18,607 |
| Control cities | 28,339 | 22,699 | 107,851 | 158,037 |

Estimation

We used the synthetic control method (SCM) developed by Abadie, et al. (2010) to estimate the impact of SSB taxes on outcomes the year the policy was put into effect and the years following. This method identifies a set of weights, where the weighted average of the control cities' characteristics and city-level outcomes in each pre-intervention year matches with the those of the intervention city. The weighted average of the control cities' outcomes is referred to as the synthetic control. The intervention effect was estimated as the difference between the observed outcome in the intervention city and the estimated outcome in the synthetic control during the post-tax phase.

To calculate the weights in the synthetic control, a symmetric and positive semidefinite matrix needs to be specified to standardize all city-level matching variables. We specified a diagonal matrix, where the entries were the inverse of the variances of the pre-intervention year outcomes and demographic summaries weighted by a scalar factor that prioritizes the predictive power of the pre-intervention outcomes (specified below). We used a standard quadratic programming approach to estimate the synthetic control weights (Goldfarb, Idnani, 1982, 1983; Turlach, Weingessel, 2019).

The success of the synthetic control methods also relies on the assumption that the pre-intervention outcomes lie within the convex hull of the control city pre-intervention outcomes. When deviations to this assumption were

© 2024 Young DR et al. *JAMA Network Open.*

encountered in our data, we considered the following transformations of the outcomes (Abadie, 2021): 1) subtracting the mean of each city's pre-intervention outcomes; 2) subtracting each city's intercept (outcome in the first year of under study); 3) subtracting each arm's intercept; and 4) first order differencing. If a transformation was needed, we chose the transformation that resulted in the best alignment between the intervention city and synthetic control outcomes in the pre-intervention period. We performed estimation and inference of the intervention effects on the transformed outcomes.

Separate SCM analyses were conducted for each pre-planned analysis (i.e., overall and subgroup analyses) and each outcome. Subgroup estimates may not sum up to the overall estimate due to the lack of constraints among separate SCM analyses.

Inference

We used the permutation test approach for inference by Abadie et al. (2010) and Bottmer et al., (2023). We applied 30 rounds of permutations. In all permutation rounds, the real treated city was relabeled as a control city and one control city was relabeled as a treated city. We further shifted the timeline by one year. Specifically, for intermediate years during the post-tax period, we shifted the timeline one year before and after the actual time. For the last year during the post-tax phase, we shifted one year and two years before the actual time. By the exchangeability assumptions in Abadie et al. (2010) and Bottmer et al., (2023) and under the null hypothesis with no treatment effect, the 30 rounds of permutations and the actual study design are a random sample of the null distribution with mean 0. The test statistic for inference is the ratio between the post-intervention root mean squared prediction error (RMSPE) and pre-intervention RMSPE (Abadie, 2021, equation 12). The test statistic is only a two-sided test since the RMSPE does not have directional information. We obtained p-values based on the permutation distribution of each of these test statistics, separately.

Although the EHR data had a massive number of patients and records, the synthetic control method is based on the sparse aggregated city-year data (44 cities by 10 or 12 years). The relatively small numbers of cities and years greatly weakened the statistical power in drawing inference. The p-value of the RMSPE test is a two-sided discrete-valued function with the step size of 0.033 since there are only 30 rounds of permutations to calculate the p-value. Thus, our sensitivity analysis has limited power to declare statistical significance for the usual p-value cutoff such as 0.01 or 0.05.

Combining findings across all intervention and control cities

To combine average intervention effects across the four tax cities, which we refer to as aggregated intervention effects, we calculated a weighted average of the four point estimates for each treated city, where the weights were defined using the average annual number of children in each intervention city (excluding 2020) (Krief, et al., 2016; Robbins, et al., 2017). Inference was performed using the permutation approach described previously, where the test statistics were the aggregated intervention effects.

Results

The **eFigure** visualizes the synthetic control analyses by tax cities and the three outcomes. The trends of all outcomes in the pre-tax phase were generally well matched between a tax city and its synthetic control. This observation suggests that the SCM was successfully implemented to the aggregated data. In the post-tax phase, the synthetic control's trend in the BMI percentile outcome was notably higher than the tax city's observed trend, suggesting a beneficial treatment effect in lowering the BMI percentile. By contrast, the post-tax trends in the obesity and obesity/overweight status outcomes did not have clear and consistent distinctions between tax cities and synthetic controls, suggesting the lack of a treatment effect on these two outcomes.

The **eTable 2** provides detailed estimates and p-values of the SCM analyses. The point estimates are generally similar to the main results. Since the SCM approach is fundamentally different from the DID approach in the main analysis, we considered that the similarity in estimates is strong evidence to the robustness in the quantitative results. P-values from the SCM are generally larger than those from the corresponding DID analyses, which is expected due to the poor statistical efficiency of the SCM.

© 2024 Young DR et al. *JAMA Network Open.*

**eReferences**

B. Han and M. A. Sidell (2024). Pseudo p-values for assessing covariate balance in a finite study population with application to the California sugar sweetened beverage tax study. arXiv:2404.09960

Lechner M. (2011). The estimation of causal effects by difference-in-difference methods. *Foundations and Trends in Econometrics*. 4(3):165-224.

McLean, R. A., Sanders, W. L., and Stroup, W. W. (1991). A unified approach to mixed linear models. *American Statistician* 45:54–64.

Conley, T.G. and Taber, C.R., 2011. Inference with "difference in differences" with a small number of policy changes. *The Review of Economics and Statistics*, 93(1), pp.113-125.

Rambachan, A. and Roth, J., 2023. A more credible approach to parallel trends. *Review of Economic Studies*, 90(5), pp.2555-2591.

Abadie, A., Diamond, A. and Hainmueller, J., (2010). Synthetic control methods for comparative case studies: Estimating the effect of California's tobacco control program. *Journal of the American Statistical Association*, *105*(490), pp.493-505.

D. Goldfarb and A. Idnani (1982). Dual and Primal-Dual Methods for Solving Strictly Convex Quadratic Programs. In J. P. Hennart (ed.), Numerical Analysis, Springer-Verlag, Berlin, pages 226–239.

D. Goldfarb and A. Idnani (1983). A numerically stable dual method for solving strictly convex quadratic programs. *Mathematical Programming*, **27**, 1–33.

S original by Berwin A. Turlach R port by Andreas Weingessel Fortran contributions from Cleve Moler dpodi/LINPACK) (2019). quadprog: Functions to Solve Quadratic Programming Poblems. R package version 1.5-8. https://CRAN.R-project.org/package=quadprog

Abadie, A. (2021). Using synthetic controls: Feasibility, data requirements, and methodological aspects. *Journal of Economic Literature*, *59*(2), pp.391-425.

Bottmer L, Imbens G, Speiss J, Warnick M (2023). A design-based perspective on synthetic control methods. arXiv:2101.09398

Kreif, N., R. Grieve, D. Hangartner, A. J. Turner, S. Nikolova, and M. Sutton (2016). Examination of the synthetic control method for evaluating health policies with multiple treated units. Health economics 25 (12), 1514–1528.

Robbins, M., J. Saunders, and B. Kilmer (2017). A Framework for Synthetic Control Methods with High-Dimensional, Micro-Level Data: Evaluating a Neighborhood-Specific Crime Intervention. Journal of the American Statistical Association 112 (517), 109–126.

© 2024 Young DR et al. *JAMA Network Open.*

**eFigure. Average outcome in each study year for each tax city and corresponding synthetic control**

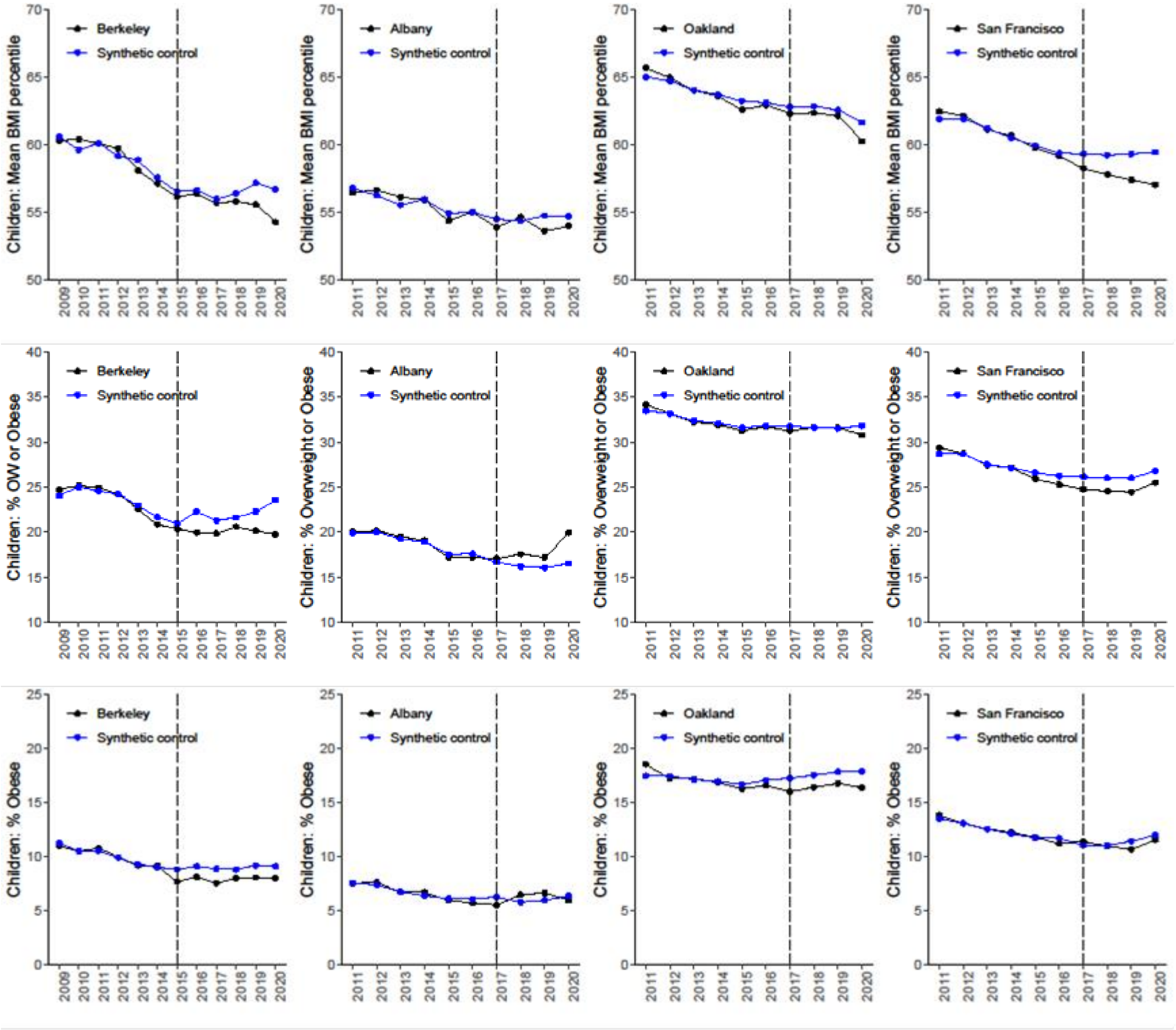


© 2024 Young DR et al. *JAMA Network Open.*

**eTable 2. Overall and subgroup intervention effects using the synthetic control method, aggregated over the 4 intervention cities with permutation-based p-values**

| | BMI percentile | | Overweight or obese (%) | | Obese (%) | |
|---|---|---|---|---|---|---|
| | **Effect** | **p-value** | **Effect** | **p-value** | **Effect** | **p-value** |
| Overall | -1.18 | 0.10 | -0.93 | 0.17 | -0.72 | 0.37 |
| Age | | | | | | |
| 2-5 | -2.88 | <0.03 | -0.18 | 0.53 | -0.10 | 0.37 |
| 6-11 | -1.17 | <0.03 | -0.59 | 0.23 | -0.23 | 0.57 |
| 12-19 | -1.09 | 0.13 | -0.81 | 0.03 | -0.38 | 0.10 |
| Sex | | | | | | |
| Female | -1.10 | 0.13 | -0.57 | 0.50 | -0.47 | 0.37 |
| Male | -1.20 | <0.03 | -0.61 | <0.03 | -0.45 | 0.13 |
| Race/ethnicity | | | | | | |
| Asian | -0.86 | 0.27 | -0.80 | 0.13 | -0.30 | 0.17 |
| Black | 0.84 | 0.33 | -0.57 | <0.03 | -0.39 | 0.08 |
| Hispanic | -0.38 | 0.23 | -0.04 | 0.90 | -0.32 | 0.63 |
| White | -1.33 | 0.20 | 0.09 | 0.87 | 0.06 | 0.50 |

p-values are based on permutation testing based on the ratio between the post-intervention root mean squared prediction error (RMSPE) and pre-intervention RMSPE (Abadie, 2021, equation 12), as described in the eMethods.

© 2024 Young DR et al. *JAMA Network Open.*
