# Supplementary material for: City-Level Sugar-Sweetened Beverage Taxes and Youth Body Mass Index Percentile
Source: JAMA Netw Open. 2024 Jul 31;7(7):e2424822. doi: 10.1001/jamanetworkopen.2024.24822 (PMC11292449; doi:10.1001/jamanetworkopen.2024.24822)
Supplement: Supplement 2. — Data Sharing Statement [file jamanetwopen-e2424822-s002.pdf]

Data Sharing Statement

Young. City-Level Sugar-Sweetened Beverage Taxes and Youth Body Mass Index Percentile. *JAMA Netw Open*. Published July 31, 2024. doi:10.1001/jamanetworkopen.2024.24822

Data
Data available: No

Additional Information
Explanation for why data not available: The data set includes health data of millions of KP members and is not available.
